# Supplementary material for: Genetic Deletion of the LINC00520 Homolog in Mouse Aggravates Angiotensin II-Induced Hypertension
Source: Noncoding RNA. 2023 May 15;9(3):31. doi: 10.3390/ncrna9030031 (PMC10204496; doi:10.3390/ncrna9030031)
Supplement: Supplementary file 1 [file ncrna-09-00031-s001.zip › ncrna-2336896-supplementary.pdf]

Supplementary Materials

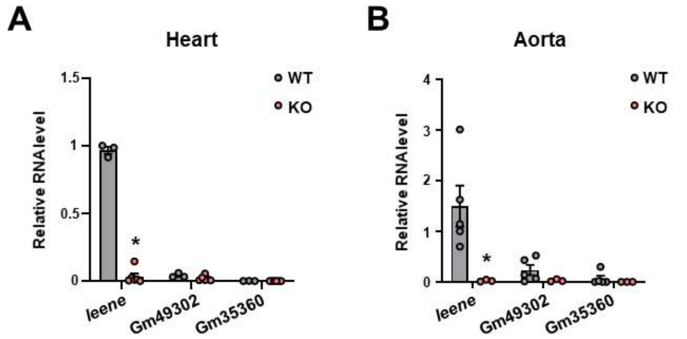

Supplemental Figure S1. qPCR of Gm41148 (leene), Gm49302, and Gm35360 in mouse heart (A) and aorta (B). 36b4 was detected as internal control. Data represent mean±SEM. *P< 0.05 compared to WT based on t test. n=3-6/group.

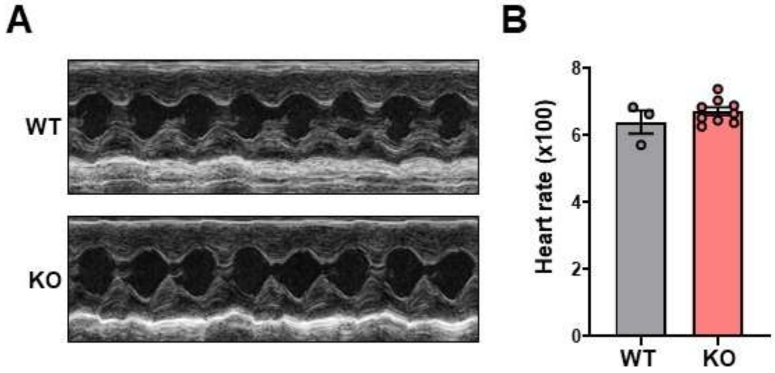

Supplemental Figure S2. Heart function in WT and KO male mice at 6 month-old at baseline. (A) Representative M-mode images of echocardiography and (B) heart rate from WT and *leene*-KO mice. n=3-9 mice per group. Data are represented as mean ± SEM.

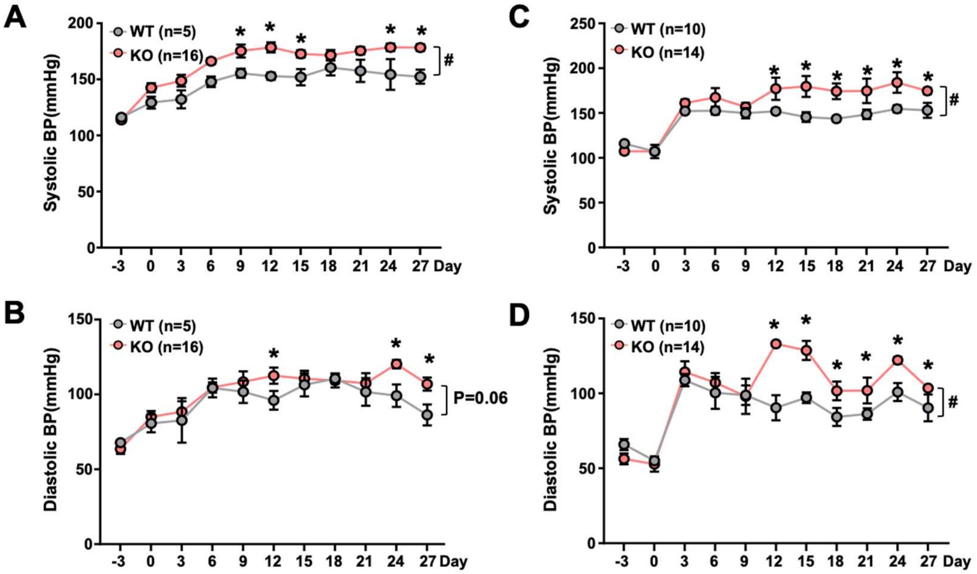

Supplemental Figure S3. BP in two other batches of mice. Systolic and diastolic BP in AngII-infused male mice of 6 month-old (A,B) and 4 month-old (C,D). n=5-16 mice per group in (A,B) and n=10-14 mice per group in (C,D). Data are represented as mean ± SEM. *P<0.05 between WT and KO mice at the same time point based on two-tailed Student's t-test. #P<0.05 between WT and KO based on repeated-measures t-test.

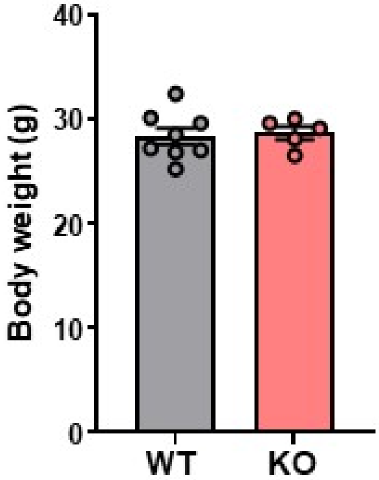

Supplemental Figure S4. Body weight of 6 month-old WT and KO male mice with AngII infusion. n=5-8 mice per group. Data are represented as mean ± SEM.

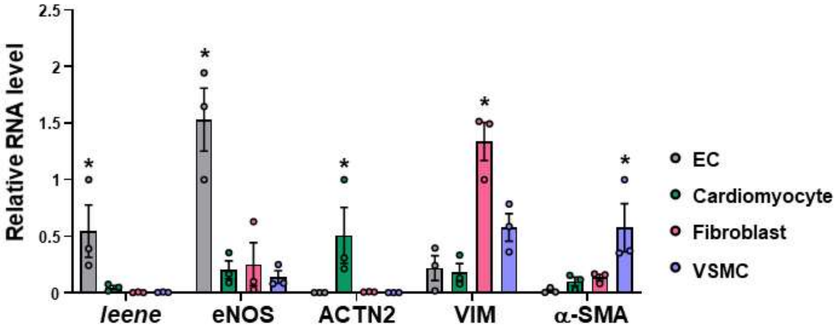

Supplemental Figure S5. qPCR of leene and cell type markers in four different cell types in WT mice. Data are represented as mean±SEM. *P< 0.05 compared among 4 cell types based on one-way ANOVA followed by Dunnett's test.

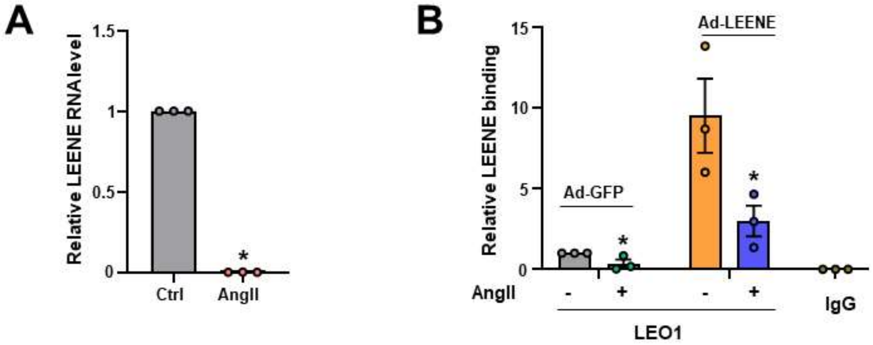

Supplemental Figure S6. AngII decreases LEENE and its interaction with LEO1. (A) qPCR of LEENE in HUVECs treated with vehicle control or AngII (100 nM) for 48h. (B) RIP-qPCR with LEO1 antibody to detect the interaction of LEENE and LEO1. The relative enrichment in Ad-GFP sample was set as 1. IgG was used as an antibody control. Data are represented as mean±SEM from three independent experiments. *P <0.05 based on t test.

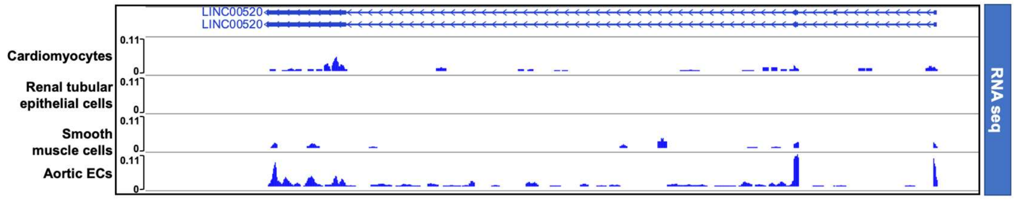

Supplemental Figure S7. LEENE transcription levels in 4 human cell types. RNA-seq data retrieved from ENCODE and Epigenome Roadmap showing LEENE RNA transcription in multiple human cell types as indicated.

Supplemental Table S1. Sequences of primers

Gene/Primer ID	Species	Assay	Sequence
36B4	mouse	RT-PCR	Forward: AGATTCGGGATATGCTGTTGGC<br>Reverse: TCGGGTCCTAGACCAGTGTTC
iNOS	mouse	RT-PCR	Forward: GTTCTCAGCCCAACAATACAAGA<br>Reverse: GTGGACGGGTCGATGTCAC
KLF4	mouse	RT-PCR	Forward: GGCGAGTCTGACATGGCTG<br>Reverse: GCTGGACGCAGTGTCTTCTC
KLF2	mouse	RT-PCR	Forward: GAGCCTATCTTGCCGTCCTTT<br>Reverse: CACGTTGTTTAGGTCCTCATCC
eNOS	mouse	RT-PCR	Forward: CTTGACCCAATAGCTGCTCAG<br>Reverse: CACCTACGACACCCTCAGTG
AGF	mouse	RT-PCR	Forward: ATGCACAGATCGGAGATGACT<br>Reverse: CATGCAGGGTCTTCTCATTCAC
ATP2B1	mouse	RT-PCR	Forward: TGAAGGAGCTGCGATCCTCTT<br>Reverse: CTGTTCCTGCTCAATTCGACT
leene	mouse	RT-PCR	Forward: TCTCACCCCTTCCTGGTACAT<br>Reverse: CCCCTTTGTCCTTCCTAGGTC
VCAM1	mouse	RT-PCR	Forward: AGTTGGGGATTCGGTTGTTCT<br>Reverse: CCCCTCATTCCTTACCACCC
BNP	mouse	RT-PCR	Forward: CTGAAGGTGCTGTCCCAGAT<br>Reverse: CCTTGGTCCTTCAAGAGCTG
COL1	mouse	RT-PCR	Forward: GCTCCTCTTAGGGGCCACT<br>Reverse: ATTGGGGACCCTTAGGCCAT
MYH7	mouse	RT-PCR	Forward: ATCAATGCAACCCTGGAGAC<br>Reverse: CGAACATGTGGTGGTTGAAG
Gm49302	mouse	RT-PCR	Forward: CTGGCACCCACTAGGATGAC<br>Reverse: AGCAAATGGTCCCTTGGGTT
Gm35360	mouse	RT-PCR	Forward: AGCCGTTGGAAAAGGGTGAA<br>Reverse: CTGAGAAGGTGCTACGGGTG
ACTN2	mouse	RT-PCR	Forward: CATCGAGGAGGATTTCAGGAAC<br>Reverse: CAATCTTGTGGAACCGCATTTT
α-SMA	mouse	RT-PCR	Forward: GACTCTCTTCCAGCCATCTTTC<br>Reverse: GACAGGACGTTGTTAGCATAGA
VIM	mouse	RT-PCR	Forward: TCCACACGCACCTACAGTCT<br>Reverse: CCGAGGACCGGGTCACATA
LEENE	human	RT-PCR	Forward: TTTCCCTCTTTGGGGTCTCA<br>Reverse: GCCCTTTGATGAGTGAGTCG
VCAM1	human	RT-PCR	Forward: GTCAATGTTGCCCCCAGAGA<br>Reverse: TTTTCGGAGCAGGAAAGCCC
eNOS	human	RT-PCR	Forward: TGATGGCGAAGCGAGTGAAG<br>Reverse: ACTCATCCATACACAGGACCC
ACTB	human	RT-PCR	Forward: CATGTACGTTGCTATCCAGGC<br>Reverse: CTCCTTAATGTCACGCACGAT
